# Effect of biocompatible nucleants in rapid crystallization of natural amino acids using a CW Nd:YAG laser

**DOI:** 10.1038/s41598-018-34356-0

**Published:** 2018-10-30

**Authors:** Shilpa Thippeshappa, Sajan D. George, Aseefhali Bankapur, Santhosh Chidangil, Deepak Mathur, Abdul Ajees Abdul Salam

**Affiliations:** 1 Department of Atomic and Molecular Physics, Manipal Academy of Higher Education, Manipal, 576 104 Karnataka India; 2 Centre for Applied Nanosciences, Manipal Academy of Higher Education, Manipal, 576 104 Karnataka India

## Abstract

Laser-induced crystallization is emerging as an alternative technique to crystallize biomolecules. However, its applications are limited to specific small molecules and some simple proteins, possibly because of the need to use high-intensity, pulsed lasers and relatively long laser irradiation time. Both these factors tend to denature biological molecules. If the laser-intensity and time required to crystallize biomolecules were to be reduced, laser-induced crystallization may well become of widespread utility. We report here the crystallization of nineteen natural amino acids by a laser-induced method in combination with one of three nucleants: aluminum, coconut coir, and peacock feather barbule. We have utilized a low-power, continuous wave (CW) Nd:YAG laser (λ = 1064 nm). The advantages of our method are (i) the use of very small laser powers (60 mW), and (ii) the ability to obtain diffraction quality crystals within a mere few seconds. For most amino acids our method yields several orders of magnitude reduction in crystallization time. The use of biocompatible nucleants like coir fibres and peacock feather barbules are novel; their non-toxic nature may find broad applicability in rapid crystallization of diverse biological molecules.

## Introduction

X-ray crystallography is an accurate and widely-used technique to determine the three-dimensional (3D) structure of proteins, DNA and ligand molecules, and is of contemporary importance in structure-based drug design^1^. After more than 100 years of research and development in X-ray crystallography, obtaining high quality crystals for diffraction studies continues to be a rate-limiting step. While getting good quality crystals of small molecules may now be relatively straightforward using the conventional slow evaporation technique, except for certain natural and synthetic molecules^2^, in the case of protein molecules the process is usually tedious and often unpredictable^3^. Therefore, developing methods to grow high quality crystals of macromolecules suitable for X-ray diffraction studies continues to be highly relevant. Extensive research has been carried out to this end and various alternative methods have, indeed, been proposed to obtain protein crystals; these include the use of electric fields^4^, ultrasound energy^5^, centrifugation^6^, high-pressure cooling^7^, introducing nucleants^8^, employing micro-batches^9^, and in recent years, by using laser light^10^.

Laser-induced crystallization has been explored in recent years but its applicability to a wide enough range of biomolecules remains largely unexplored. In early studies, Garetz *et al*.^10^ succeeded in crystallizing urea by focusing a 1064 nm wavelength, Q-switched, pulsed Nd:YAG laser on a supersaturated (11.5–13.5 M) urea solution using laser fluence over the range 50–250 MW/cm^2^. It was demonstrated that such laser pulses, of nanosecond duration, crystallized urea in shorter time than conventional methods. Subsequently, non-photochemical laser-induced nucleation (NPLIN) was used to crystallize different polymorphs of urea by using linearly and circularly polarized laser light^11,12^. Shorter (femtosecond) pulses were also used to crystallize lysozyme, glucose isomerase (GI), ribonuclease H (RNase H), and trypanosoma brucei prostaglandin F2 synthase (TbPGFS) proteins^13^.

Recently, we have crystallized both small biomolecules and a protein (lysozyme) using a femtosecond laser (λ = 800 nm, 60 fs pulse duration, 5.2 MHz repetition rate, 300 mW average power)^14^. This work showed that laser-induced crystallization is highly useful in obtaining crystals of small molecules, including chalcone compounds, which are otherwise difficult to crystallize by conventional crystallization methods^14^. We have also used simple but highly effective nucleants with a CW Nd:YAG (λ = 1064 nm) laser to crystallize NaCl, KCl, glycine, aspartic acid, and histidine amino acids, and lysozyme protein^15^. This work has provided evidence that laser-facilitated, nucleants-assisted crystallization may offer the important advantage of very low incident laser power and the consequent non-alteration of biomolecule structures.

The use of external nucleants to obtain protein crystals is not new in X-ray crystallography^16^. McPherson and Shlichta first proposed heterogeneous materials as nucleants for the epitaxial growth of protein crystals^17^. In the last three decades, several nucleants have been tested, and a recent report has cogently summarized the nucleants proposed so far to crystallize protein molecules^18^. Recently, porous materials like bioglass and metal-organic frameworks (MOFs) have been proposed as universal nucleants^19–22^. These porous nucleants have produced crystals of several proteins, including membrane proteins; they have also increased the success rate of crystallization due to the combined effect of the diffusion-adsorption mechanism^19^.

We recently reported the use of aluminum nucleants in conjunction with a laser to crystallize glycine, aspartic acid, and histidine amino acids on time scales as short as a few seconds^15^. In comparison with other nucleants, aluminum yielded significant time reduction and produced consistently positive results. Based on these results we have now extended our scope by crystallizing nineteen natural amino acids using aluminum wire as a nucleant. We have also discovered that two naturally available porous materials - coconut coir and peacock feather barbule - act as efficient nucleants. These biocompatible nucleants are porous, non-toxic, and low cost. The efficacy of these three nucleants is tested by crystallizing nineteen amino acids within ~30 s after application of only 60 mW laser power from a CW Nd:YAG (λ = 1064 nm) diode laser. We find that, in comparison with conventional crystallization, the combination of laser irradiation with one of our three nucleants yields amino acid crystallization on time scales that are up to ~84 times faster. Our results suggest the possibility that some biocompatible porous nucleants may be helpful in reducing both crystallization time and the laser power required to crystallize biological molecules. The quality of our rapidly grown crystals has been verified by collecting cell parameters using single-crystal X-ray crystallography. A possible mechanism, as well as the general applicability of nucleant-assisted laser-induced crystallization, is discussed.

## Results and Discussions

Peacock feather barbules may have blue, green or brown colour. Initial experiments were conducted to choose the appropriately coloured peacock feather barbule. Glycine (200 mg/ml) and alanine (50 mg/ml) crystals were grown with blue, green and brown barbules. We observed that irrespective of the colour of the barbules, laser-induced nucleation could be achieved on almost similarly short time scales: ~3 s and ~5 s for glycine and alanine, respectively. Structurally, there is no difference between these colour barbules, and all three are photonic crystals made up of melanin rods connected by keratin. The lattice structures of the blue, green barbules are nearly square while the brown ones are rectangular in shape and devoid of an air hole array between melanin layers nearest to the surface. The difference arises mostly in the construction of the rod spacing (lattice constant) and some melanin rod layers along the normal to the cortex surface^23^. Scanning electron microscopy results indicate that the lattice constant for blue (~140 nm), green (~150 nm) and brown (~150 and 180 nm) barbules are nearly identical^23^. Since the green barbule has the median lattice constant, we chose this type for further experiments. The results that we obtained are summarised in the following, based on the solubility of the amino acids as well as on their properties. Laser-induced crystallization experiments were conducted under identical conditions with three nucleants, aluminum, peacock feather barbule, and coconut coir; the respective set of results are hereafter referred to as Laser-AL, Laser-PF, and Laser-CO, respectively.

### Freely soluble amino acids

This group has four amino acids: glycine, lysine, serine and proline. Glycine molecules needed 1720 s to crystallize without any nucleant or laser exposure. When a nucleant was added to a glycine droplet, a slight reduction in time was observed. Control-AL, control-PF, and control-CO took 1320 s, 1360 s, 1440 s respectively. On average, our control experiments (without laser light) needed 1460 s to form the first crystal. When similar experiments were carried out under laser irradiation, significant time reduction was observed. Laser-AL took 3.3 s whereas both Laser-PF and Laser-CO took 5 s to induce nucleation. On average, laser-irradiated glycine samples with nucleants needed ~4 s, which is 329 times faster than in our control experiments (Table 1).

**Figure 1:**
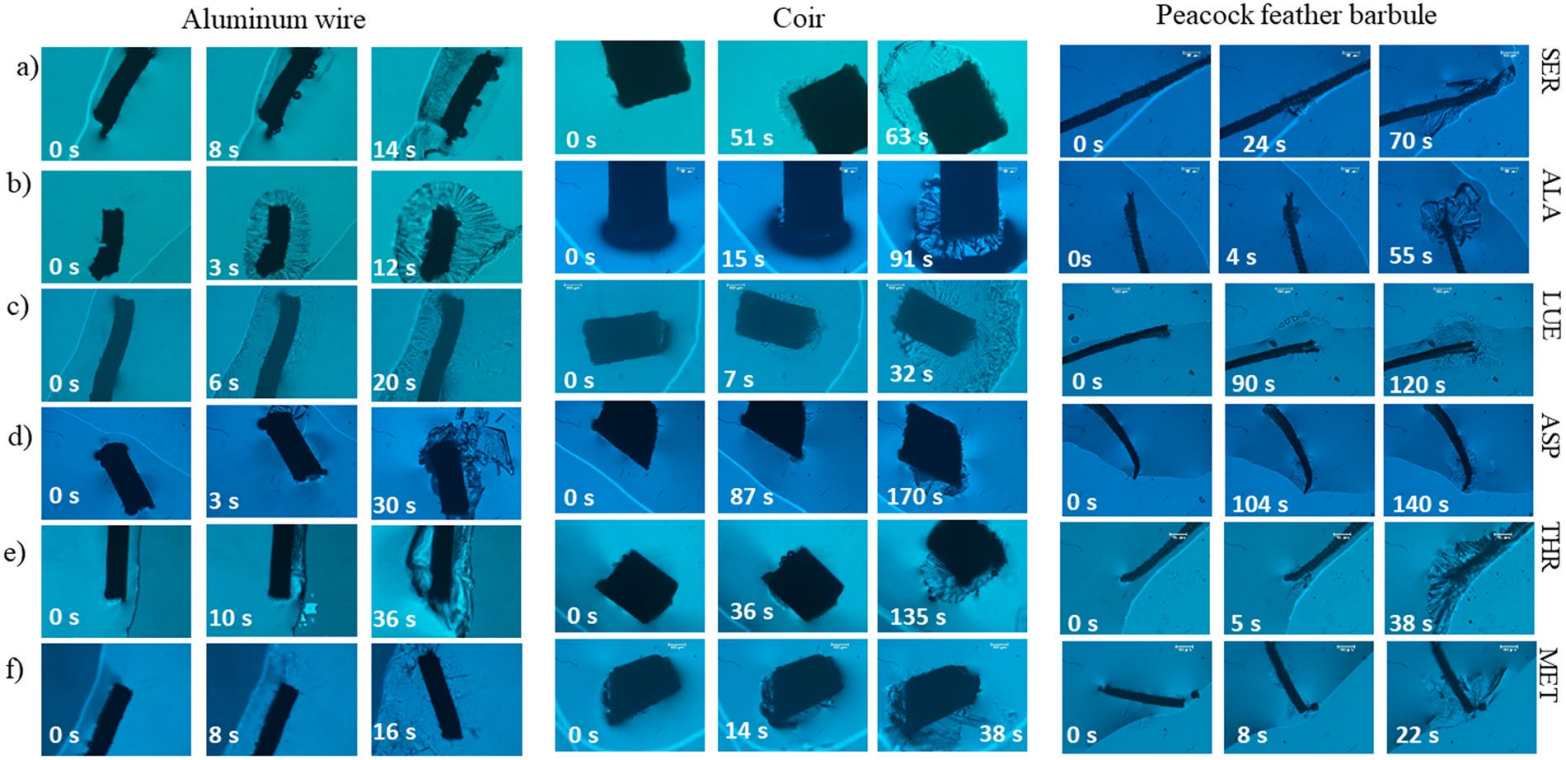
Representative time evolution of amino acid crystals upon irradiation by CW Nd:YAG laser light of typical power 60 mW. Rows (**a**–**f**) depict, respectively, crystals of SER, ALA, LUE, ASP, THR, and MET.

In our previous report^15^, we had reported crystallization of glycine molecules ranging from 1M (75 mg/ml) – 3M (225 mg/ml) concentration using different nucleants. Aluminum wire, as well as aluminum particles, took more or less similar times (3 s) in those studies, which is comparable to the values measured in our current experiments. Lysine is another essential amino acid that we sought to crystallize. In our control (laser-less) experiments, it took an average of 3380 s to yield the first crystal of lysine. Once again, there was no significant difference observed upon inclusion of nucleants in our control experiments. On the other hand, laser-irradiated samples with nucleants provided lysine crystals within only 17 s, which is 197-fold faster than in the control experiments. Serine and proline took an average of 22 s and 15 s, respectively, which is 170 and 252 times faster, respectively, than in control experiments. Representative time evolution of serine crystals under laser irradiation is depicted in Fig. 1 and a real-time movie is presented as Video SV1 in Supplementary Information.

In the present study, we have introduced two biocompatible nucleants, peacock feather and coconut coir, which are naturally available, cost-effective and inert. We also used aluminum as a nucleant, and we found that it yields nucleation faster than coir and peacock feather barbule under laser-irradiation (Table 1). However, there may be concerns that aluminum interacts with biomolecules, especially when the biomolecules have polar solvent present in an acidic environment (aluminum may, then, be oxidized). In this scenario, biocompatible nucleants were deemed more appropriate for our crystallization experiments. Overall, crystallization of glycine, lysine, proline and serine under laser-irradiation with nucleants produced crystals within ~15 s, a factor of 211 times faster than in control (laser-less) experiments. This remarkable reduction in time motivated us to crystalize other groups of amino acids.

**Table 1:** Time taken (s) for crystallization of amino acids. Laser*: average time taken for laser-irradiated samples rounded to the nearest integer; Control**: average time taken for control (laser-less) experiments.

Amino acid	Laser-AL	Laser-PF	Laser-CO	Laser*	Control-NN	Control-AL	Control-PF	Control-CO	Control**	Fold
Freely soluble amino acids										
Gly	3.3 ± 0.3	5 ± 0.8	5 ± 1.5	4	1720 ± 106	1320 ± 60	1360 ± 53	1440 ± 92	1460	329
Lys	4.5 ± 0.5	25 ± 1	22 ± 3	17	3600 ± 92	3660 ± 35	2900 ± 280	3360 ± 193	3380	197
Pro	11.3 ± 1.8	20 ± 8	13 ± 1.5	15	4120 ± 105	4240 ± 80	2560 ± 160	3960 ± 13	3720	252
Ser	6.3 ± 0.6	7.5 ± 0.5	51.5 ± 1	22	3780 ± 35	3760 ± 20	3760 ± 5	3540 ± 250	3710	170
Average	6	14	23	15	3305	3245	2645	3075	3068	211
Soluble amino acids										
Ala	3.3 ± 0.3	4.3 ± 0.3	15.3 ± 1.5	8	3760 ± 20	3540 ± 6	3660 ± 20	3600 ± 13	3640	477
Val	4.3 ± 0.3	8 ± 1	26.3 ± 1.7	13	2220 ± 60	1800 ± 60	1780 ± 53	1660 ± 20	1865	145
Met	3.3 ± 0.3	6.3 ± 0.3	13 ± 0.6	8	540 ± 20	520 ± 20	520 ± 20	500 ± 20	520	69
Thr	7.5 ± 2.5	4.5 ± 0.5	37.7 ± 4.7	17	3460 ± 174	3120 ± 20	4000 ± 20	3060 ± 60	3420	206
His	84 ± 4.6	233 ± 11	110 ± 15	142	4740 ± 104	4100 ± 111	4420 ± 100	3980 ± 20	4310	30
Ile	8.7 ± 0.3	32 ± 3	12.5 ± 0.5	18	620 ± 20	560 ± 40	540 ± 60	540 ± 60	565	32
Cys	7 ± 0.6	4.5 ± 0.5	18 ± 11.5	10	2320 ± 20	1920 ± 35	2060 ± 122	1680 ± 35	1995	203
Average	17	42	33	31	2522	2223	2426	2146	2329	76
Sparingly soluble										
Asn	10 ± 1	95 ± 2.5	75 ± 11	60	2800 ± 231	2800 ± 106	3280 ± 144	2940 ± 92	2955	49
Phe	5 ± 1	12 ± 7	49.5 ± 2.5	22	860 ± 20	760 ± 80	800 ± 40	640 ± 40	765	35
Leu	6 ± 0.5	88 ± 11	8 ± 0.6	34	520 ± 20	460 ± 40	500 ± 40	440 ± 20	480	14
Gln	14 ± 1.6	24.5 ± 1	12.7 ± 0.3	17	4200 ± 183	3620 ± 231	3160 ± 160	3640 ± 80	3655	214
Average	9	55	36	33	2095	1910	1935	1915	1964	59
Slightly soluble										
Glu	24 ± 0.5	28 ± 1.7	84.5 ± 1.7	46	1480 ± 180	1440 ± 20	1320 ± 60	1380 ± 20	1405	31
Trp	20.7 ± 0.6	8.5 ± 1.5	49 ± 3.2	26	3760 ± 53	3380 ± 20	3600 ± 60	3380 ± 20	3530	135
Tyr	8 ± 1.4	21 ± 1	81 ± 2	37	2840 ± 140	2900 ± 100	2760 ± 60	2840 ± 72	2835	77
Asp	2.5 ± 0.5	85 ± 0.8	86 ± 2.5	58	3800 ± 20	3640 ± 203	3460 ± 140	3380 ± 120	3570	62
Average	14	36	75	42	2970	2840	2785	2745	2835	68
Average of all 19 AA	12	37	41	30	2692	2502	2444	2419	2514	84

The column marked “Fold” quantifies the time acceleration factor achieved in our laser-induced rapid crystallization.

### Soluble amino acids

This group has seven amino acids: [L-alanine (Ala), L-valine (Val), L-methionine (Met), L-threonine (Thr), L-histidine (His), L-isoleucine (Ile) and L-cysteine (Cys)]. Overall, the control experiment (without recourse to laser irradiation) yielded crystals within ~2329 s. In contrast laser-assisted experiments yielded crystals within ~31 s, which is 76 times faster than in control experiments. In case of Laser-AL, histidine (His) took ~84 ± 4.6 s while the remaining amino acids in this group were crystallized within ~9 s. Similarly, Laser-PF took ~233 ± 11 s to produce His crystals, and Ile took ~32 s. Except for His and Ile, the remaining amino acids were crystallized within ~8 s using Laser-PF. In Laser-CO, His took relatively longer time (110 ± 15 s) compared to all other amino acids (see Table 1). The longer time taken to crystallize His appears to be consistent with earlier (non-laser) experiments, like those of Alabanza *et al*. where 238 minutes were required to obtain initial crystals of His at room temperature; the corresponding times for Ser and Val were 60 min in each case in their experiment^24^.

In partial summary, the Laser-AL method needed ~17 s to crystallize all seven amino acids. Laser-PF and Laser-CO needed ~42 s and ~33 s, respectively, a factor of two longer than the time required in Laser-AL but, nevertheless, still ~58- and ~65-fold faster than the time required in laser-less control experiments (Table 1). Overall, our laser-induced method yielded crystals within about 31 s, which is ~76-fold faster than in laser-less control experiments. A representative time evolution of Ala crystals under laser irradiation is depicted in Fig. 1. The real time rapid growth of alanine crystals when 1064 nm laser was incident on a peacock feather barbule is shown in real time in Video SV2 in the Supplementary Information.

### Sparingly soluble amino acids

This group has four amino acids: L-asparagine (Asn), L-phenylalanine (Phe), L-leucine (Leu), and L-glutamine (Gln). In control experiments, Leu crystals appeared in relatively short time (~480 s), followed by Phe (~765 s), Asn (~2955 s), and Gln (~3655 s). On average, the control (laser-less) experiments yielded crystals within ~1964 s. In contrast, Laser-AL yielded Phe and Leu crystals within ~5 ± 1 s and 6 ± 0.5 s, followed by Asn (10 ± 1 s) and Gln (14 ± 1.6 s). In case of Laser-PF, once again Phe crystallized faster (12 ± 7 s) than the remaining three amino acids, Gln (24.5 ± 1 s), Asn (95 ± 2.5 s), and Leu (88 ± 11 s) whereas in Laser-CO, Leu molecules yielded crystals very fast (8 ± 0.6 s); Gln, Phe, and Asn crystals appeared within 12.7 ± 0.3 s, 49.5 ± 2.5 s, and 75 ± 11 s, respectively. Representative time evolution of Leu crystals under laser irradiation is also depicted in Fig. 1. Overall, on an average, the minimum time required to achieve nucleation under laser irradiation was 5 ± 1 s and the maximum was 95 ± 11 s; the laser-irradiated molecules crystallized 59 times faster than in control (laser-less) experiments (Table 1). The real time rapid growth of Leu crystals upon 1064 nm laser irradiation in the presence of coir is shown in Video SV3 in the Supplementary Information.

### Slightly soluble amino acids

This group comprises four amino acids: L**-**Glutamic acid (Glu), L-aspartic acid (Asp), L-Tryptophan (Trp), and L-Tyrosine (Tyr). Crystals were obtained within ~2835 s in control (laser-less) experiments. In laser-AL, the first crystals appeared within ~2.5 ± 0.5 s for Asp, followed by ~8 ± 1.4 s for Tyr, ~20.7 ± 0.6 s for Trp, and ~24 ± 0.5 s for Glu. In case of laser-PF and Laser-CO, the first crystals occurred within ~86 s for all these amino acids (Table 1). A representative time evolution of Asp crystals under laser irradiation is shown in Fig. 1. Overall, the laser-induced amino acids crystallized ~68 times faster compared to the control experiments; real time rapid growth of Asp crystals upon irradiation by 1064 nm laser light on Al wire is shown in Video SV4 in the Supplementary Information.

### Results based on amino acid properties

To understand the crystallization behaviour on the basis of polarity, we have bifurcated our results into polar and non-polar groups. Gly, Cys, Ser, Thr, Tyr, Asn, Gln, Asp, His, Lys, and Glu are in the polar group while the remaining eight amino acids, Ala, Val, Leu, Ile, Phe, Trp, Met, and Pro, comprise the non-polar group. We found that the polar amino acids needed an average of ~16 s, ~48 s, and ~53 s to yield crystals in laser-AL, laser-PF, and laser-CO conditions, respectively. Correspondingly, the non-polar amino acids were found to require ~8 s, ~22 s and ~23 s for laser-AL, laser-PF, and laser-CO, respectively. In general, laser-induced crystallization yielded crystals faster in the non-polar amino acid group than for the polar amino acids (see Tables ST1 and ST2 in the Supplementary Information). In control (laser-less) experiments polar amino acids took ~2970 s to produce the first crystals, whereas non-polar amino acids yielded crystals within ~1886 s. In contrast, laser-induced crystallization required only ~39 s and ~18 s to produce the first crystals of polar and non-polar amino acids, respectively. Representative time evolution of polar Thr and non-polar Met crystals under the irradiation of laser is shown in Fig. 1.

In terms of charged amino acids, the positively charged amino acids, His and Lys, required an average of 142 s and 17 s, respectively, to yield the first crystals; the negatively charged amino acids, Asp and Glu, took an average of 58 s and 46 s, respectively to yield the initial crystals in all our laser-induced crystallization experiments. Both the negatively charged amino acids we used are slightly soluble and tend to crystallize at almost the same time under laser irradiation, whereas we found no correlation between the time taken to produce the first crystals in positively charged amino acids. The results suggest that the charged amino acids need more time to crystallize in comparison with uncharged amino acids (see Tables ST1 and ST2 in the Supplementary Information).

It should be noted that in some of the amino acids - like Gln, Phe - we observed both formation and dissolution of crystals; both seem to be prevalent phenomena^15,25^.

Though laser-induced crystallization is beginning to be recognized as a promising technique to crystallize biomolecules, very few amino acids (L-His, L-Phy, and L-Gly) have been crystalized using this technique so far^26–28^. Alabanza *et al*. have crystallized five amino acids (L-Thr, L-His, L-Leu, L-Ser, and L-Val-HCl) using metal-assisted and microwave-accelerated evaporative crystallization (MA-MACE). The MA-MACE method reduced the crystallization time 8-fold compared to the room temperature method^24^. In this study, Leu crystals were obtained faster (10 min) than the remaining Val (12 min), Ser (20 min), His (60 min), and Thr (60 min) crystals. Compared to this study, our present work has yielded crystals on much faster times for all amino acids. In the present experiments, the first crystals appeared in 3 minutes 50 seconds in the case of His using laser-PF; this is 65-fold faster than what has been reported using the MA-MACE method^24^.

Earlier work on growth of crystals of small molecules and amino acids crystals using laser-induced crystallization has relied on high power lasers for irradiation. In such studies, the crystallization targets are usually dissolved in either D_2_O or aged solutions are used to obtain crystals upon laser irradiation. In our previous report, we have summarized the results of laser-induced crystallization with the pertinent literature^15^. We have also shown that use of nucleants in laser-induced crystallization drastically reduces both the laser intensity that is required as well as the overall crystallization time. The current results are in good accord with this and have, moreover, yielded crystals faster than any other report^15^. Hiroshi and coworkers have reported crystal growth of Phe using laser-induced trapping in both H_2_O and D_2_O^29^. Phe dissolved in H_2_O and D_2_O solutions yielded clusters of crystals in 250 s and 600 s, respectively^29^, which is ~12 to 30 times slower than the time taken in our current experiments. Sun *et al*. have crystallized His using a Q-switched, pulsed Nd:YAG laser at 532 nm wavelength^26^; they observed crystal formation after three days. In stark contrast, we obtained His crystals within 142 s in the present study. Thus, reduction in crystallization time as well as use of less laser power (60 mW) clearly highlights the advantages of using nucleants in laser-induced crystallization.

### Effect of nucleant position in laser-induced crystallization time

We noted that the time required for observation of the first crystal varied somewhat, and we have been unable to discern any simple pattern for the variation we observed in the course of our measurements. One reason for the time variations and lack of apparent pattern may be the exact spatial position of the nucleant near the air/solution interface. The air/solution interface is an essential factor to form crystals in our laser-induced crystallization technique^28,30^ and precise and reproducible positioning of the nucleant vis-à-vis this interface is difficult to achieve. The spatial positions of the nucleants (especially the much lighter PF and CO) often fluctuated with respect to the air/solution interface, thus producing the time variations observed for the same amino acids under similar conditions. To further probe this postulate of ours, we carried out the following additional experiments. We took Gly (200 mg/ml) and Ala (50 mg/ml) amino acids with aluminum as nucleant. Initially, we placed aluminum within the centre of a 30 μl droplet. A laser beam (60 mW) passing through a 10x microscope objective was used to irradiate the nucleant. No nucleation was observed even after 10 minutes of laser-exposure. Even upon increasing the laser power to 200 mW there was no nucleation observed. The experiment was repeated by positioning the aluminum near the periphery of the droplet in the vicinity of the air/solution interface. When a 60 mW laser beam was focused on the nucleant, initial crystals are obtained for Gly within ~3 s and Ala within ~5 s. The reason for the absence of crystal formation in the first case may be ascribed to the suppression of evaporation at the centre of the droplet compared to that at the periphery of the droplet. Also, due to the non-uniform distribution of solute molecules on the boundary of the droplet, we would expect faster evaporation at the periphery of the droplet. Since evaporation of the solvent is more at the edges, normal evaporation as well as laser heating induces convective flow within the droplet and this increases the solute concentration more at the edges than in the middle. In addition, non-uniform evaporation leads to formation of surface gradients, causing Marangoni flow, which would distribute incident laser energy unevenly, giving rise to temperature gradients and consequent mass flow. Over a large number of measurements, different spatial positions of PF and CO nucleants also yielded the same results: when the nucleant was located near the periphery of the liquid droplet, fast initial crystallization was observed.

Does the size of the droplet have an effect on the above-noted sensitivity to location of the nucleant? We have recently explored droplet size variations^15^, covering the range 5–30 μl. No size dependence was noted: nucleants located near the periphery of a droplet, of whatever size, yielded the fastest crystallization.

### Mechanism

From the literature as well as our previous studies, it seems likely that bubble formation plays an essential role in crystal formation in laser-induced crystallization^15^. In the current experiments, we observed formation of fairly large bubbles for several amino acids, such as Ala, His, Ser, and Pro, although formation of micro- and nano-bubbles, a ubiquitous phenomenon, was difficult to observe under our experimental conditions^15^. Upon collapse of relatively large bubbles, small initial crystals were seen to move away from the laser focal point. Video SV5 in Supplementary Information is a real-time movie clip showing bubble formation and collapse during laser exposure of alanine in the presence of aluminum nucleant. Bubble formation in this movie clip is mostly beneath the aluminum wire and initiates crystallization. Upon reaching large enough size, the bubbles collapse and the associated violence leads to break-up of the crystal. In our experiment, when the laser was focussed on the nucleant, there is localized rise in temperature in the region where the nucleant is located. As a result, the solution becomes supersaturated and this helps initiate the nucleation. The bubbles adsorb the solute molecules on their surface and this enhances the localized solute concentration around the air bubble-host liquid interface that, in turn, helps initiate nucleation followed by faster crystal growth. In our previous report, aluminium was seen to give rise to fast nucleation: 3 M glycine molecules were crystallized within 4 ± 1 s. In our current experiments, a similar trend was also observed for several amino acids (Table 1).

**Figure 2:**
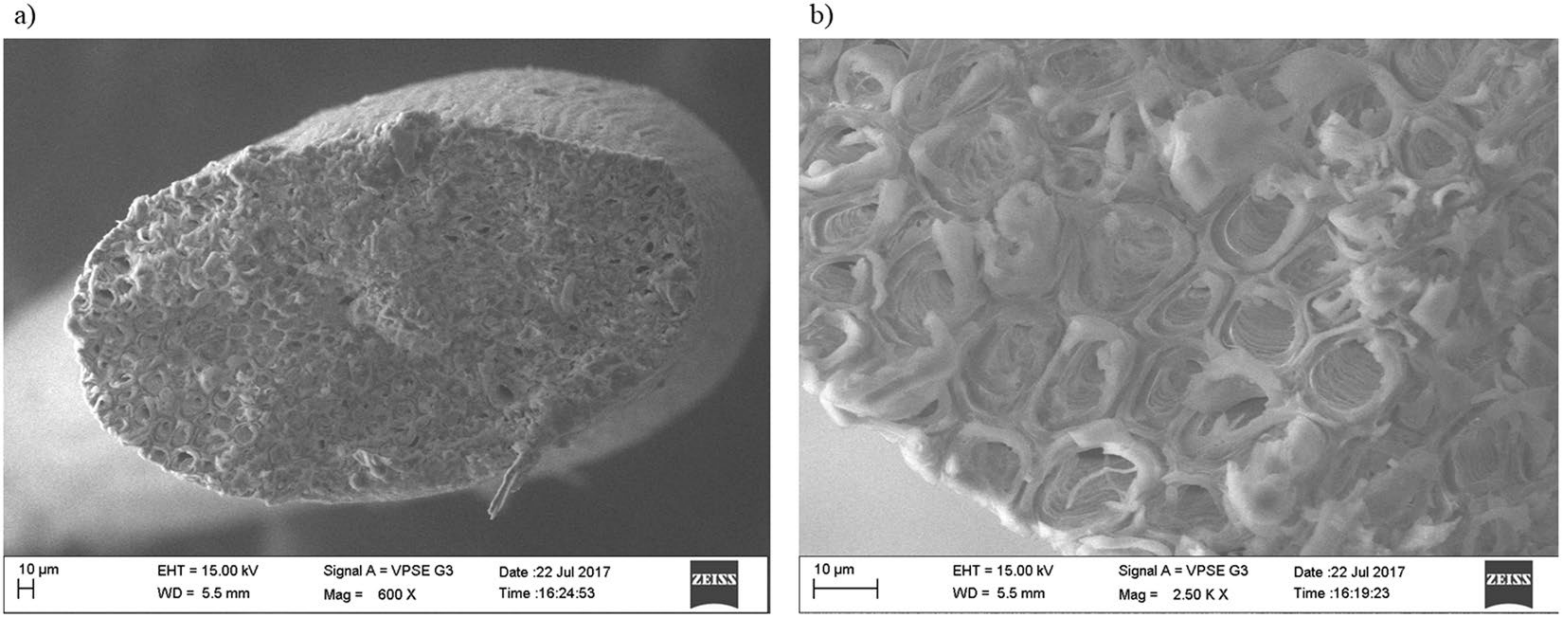
Scanning electron microscopy images of coir at (**a**) 600X magnification and (**b**) 2500X magnification.

In the case of coir nucleant, Scanning Electron Microscope (SEM) images were taken to understand the effect of the porous nature of this material and to determine the pore size. The SEM images confirmed that the coir we used was, indeed, porous in nature with regular pore size of ~10 µm (Fig. 2). Peacock feathers are photonic crystals, and SEM images taken by Zi *et al*. have shown typical pore sizes of 2–10 nm^23^. This porous nature seems to provide an advantage in crystallization of biomolecules. Recent reports have shown that porous materials (like porous silicon with diameter 5–10 nm) appear to induce nucleation even in metastable condition^31^. Nanev *et al*. have recently shown that engineered mesoporous bioglass with pore size in the range 2–10 nm increased the success rate of crystallization in the case of some membrane proteins^31^. It is also worth mentioning that pore size may be directly proportional to the size of the biomolecules^19^. Page and Sear have proposed a model explaining the crystallization of biomolecules in porous materials^32^. Initially the pore walls attract the molecules to be crystallized and nucleation is initiated within the pores. Thereafter, the crystal starts to grow inside the pores, fills the entire pore volume such that, at later times, crystal growth has to start occurring outside the pores. The underlying mechanism is two-fold: (i) trapping the biomolecules inside the pores, which increases the solute concentration locally, and (ii) free energy-driven adsorption of biomolecules at pore walls. In comparison with control, laser-induced crystallization is approximately 62 times faster in case of Laser-PF and Laser-CO; this is due to the faster diffusion of solute molecules inside the pore caused by the temperature rise. This gives rise to the rapid nucleation that we observe. The crystallization time difference observed between peacock feather barbule and coir arising from the different pores size and surface of the nucleants. Thus, the rapid nucleation observed in the current experiment is likely to be a combined effect of bubble formation due to laser heating, the thermal conductivity of the nucleant, and the porosity of the nucleant.

### Characterization of crystals

To assess the quality of our rapidly grown crystals, we measured cell parameters using single crystal x-ray diffraction (XRD). The cell parameters for the amino acids Gly, Ala, Thr, Cys, Asp, Glu, Ser, and His were determined and found to be in reasonable accord with reported structures available in the Cambridge Structural Database (see Table ST3 in the Supplementary Information). By way of illustration, in Table ST4 (Supplementary Information), we present data collection statistics and structure refinement for single crystals of C_3_H_7_NO_2_S cysteine molecule grown in the present experiments. Even though this crystal was grown within one 30 μl droplet, and the crystal size less was than 0.1 mm, agreement with reference data is encouraging.

## Summary

We have demonstrated rapid crystallization of 19 natural amino acids utilizing laser-induced crystallization in conjunction with aluminum and two biocompatible nucleants, coconut coir and peacock feather. Our crystallization method has used very low values of incident laser power (60 mW). The time taken to crystallize the amino acids has been measured to vary over the range 4–233 s, faster than that hitherto reported using conventional crystal growth techniques. Though the initial crystallization is rapid, subsequent crystal growth away from the laser focal zone seems to proceeds at rates that are essentially the same as in conventional crystal growth techniques. However, further work is required to obtain quantitative insights. We note that in our nucleant-assisted laser-induced crystallization, the three-dimensional amino acid structures remain essentially unaltered, as verified by measuring the cell parameters of crystals using single crystal XRD (Tables ST3 and ST4 in Supplementary Information; the latter Table also presents our results of crystal structure determination of Cys). Use of both aluminum and the naturally available porous materials as nucleants are novel and may find broad application in laser-induced crystallization. Similar natural porous and fibre materials with different pore size may also prove to be useful for both conventional and laser-induced crystallization. The requirement of high laser power has hitherto prevented wide scale deployment of laser-induced crystallization as an alternative technique to crystallize biomolecules. Our results on crystallization of amino acids – the building blocks of proteins – have been obtained using a low-power, CW laser and may provide an impetus to further explore the possibility of using nucleant-assisted laser-induced crystallization to provide a seed for protein molecules that have hitherto proved to be difficult to crystallize.

## Materials and Methods

Amino acids were purchased from Sisco Research Laboratory (Mumbai, India) and used as received. Ultrapure water (18.3 MΩ), obtained using a Millipore Milli-Q purification system, was used to prepare aqueous amino acid solutions. The solutions were heated to 40 ^o^C and incubated at that temperature for 10 minutes; the solutions were then slowly cooled to room temperature. Aluminum wire (thickness 0.3 mm and length 0.5 mm), coconut coir (thickness 0.4 mm and length 0.5 mm) and peacock feather barb (thickness 0.1 mm and length 0.4 mm) were individually used as nucleants. The role of aluminum as a nucleant in laser-induced crystallization was explained in details in our previous report^15^ but its use has now been extended to a larger set of molecules. Coir fibres are spongy; their major chemical constituents are cellulose (44%) and lignin (36%). There are two types of coconut fibres, brown and white. Brown fibres are thick, stiff and have high physical strength compared to white fibres^33^. The coconut fibres also have high water and salt absorbency. The coir fibre is a hollow structure comprising elementary fibres formed out of multiple cellulose-lignin cell wall layers with high porosity (22–30%)^34^. For the current experiment, brown coir fibres were mechanically extracted from the outer shells of coconuts and used as nucleants. In peacock feathers, each feather has a central rigid stem lined on both sides by a row of smaller barbs. Each barb is then bordered on both sides by rows of even tinier barbules; one such barbule was taken and placed on the glass coverslip. Moreover, we have used peacock feather barbules obtained from the eye pattern, which are blue, green and brown in colour.

**Figure 3:**
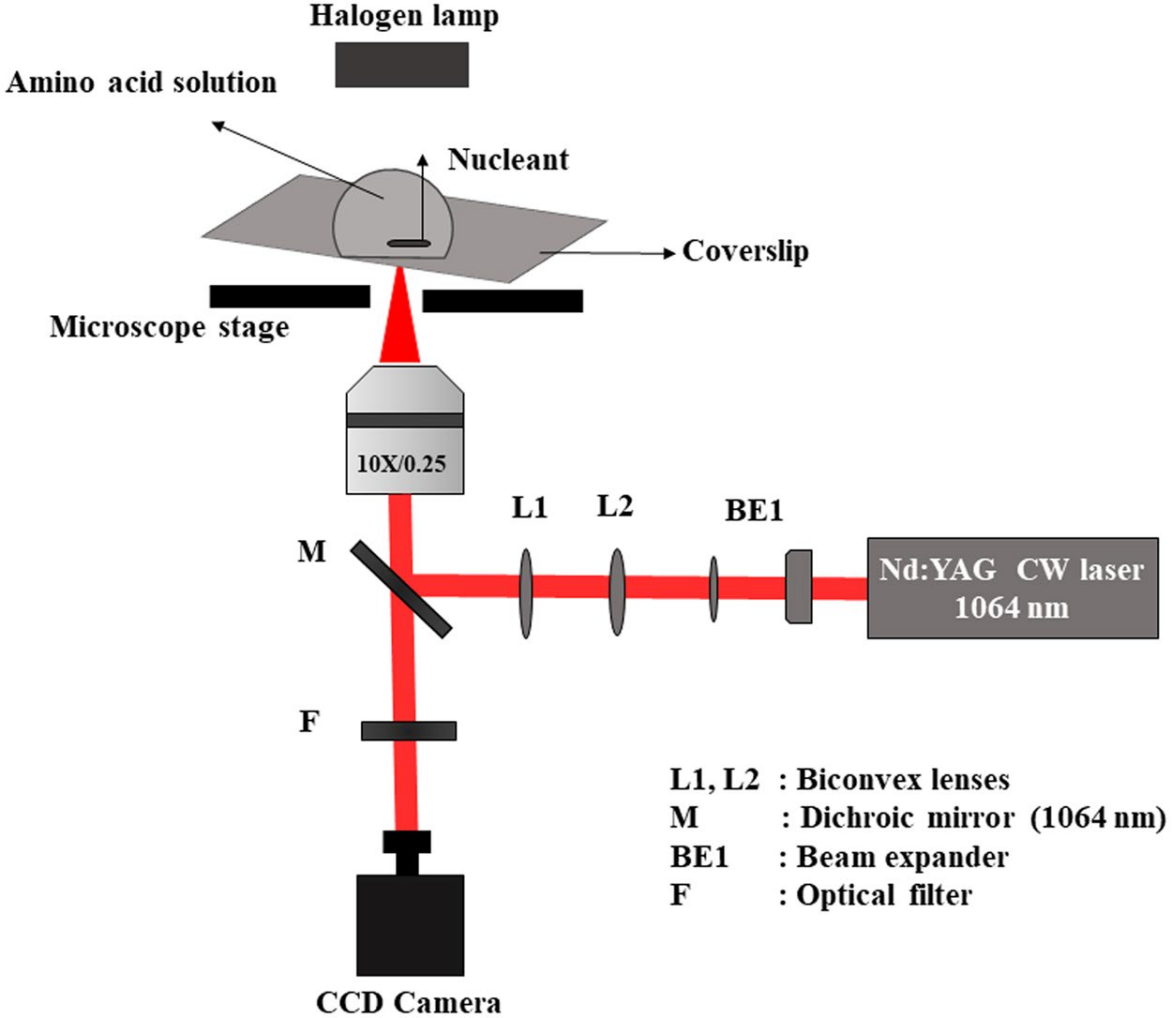
Laser-induced crystallization set-up.

A fixed volume (30 μL) of freshly prepared amino acid solution was pipetted onto a blank glass slide with the nucleant, taking care that the nucleant was entirely submerged in the amino acid solution. The coverslip was inserted on to an inverted microscope. Our experimental setup has been discussed in detail earlier^15^, but for completeness we present the schematic features of our experimental set-up in Fig. 3.

### Optical and microscope setup

A CW Nd:YAG laser (λ = 1064 nm) was used as a source of excitation. The laser power could readily be tuned over the range 1–520 mW by varying the diode current. The laser beam was expanded using a manual beam expander (BE1) to a size of 9 mm. A dichroic mirror (M) with high reflectivity at 1064 nm was used to couple the expanded laser beam via a 1:1 telescopic arrangement to the back aperture of the microscope objective (10X, NA = 0.25). A CCD camera (Nikon DS-Fi1c, Japan) was attached to one of the exit ports of the microscope, enabling visualization and recording of the crystallization process at 50 interlaced frames per second. Manipulation of focusing inside the sample was achieved using a controllable, linear x–y translational stage (Fig. 3)^35^.

Based on their solubility in water amino acids are classified into four groups^36^: (i) freely soluble, (ii) soluble, (iii) sparingly soluble, and (iv) slightly soluble. The freely soluble group has four amino acids: glycine (200 mg/ml), L-serine (250 mg/ml), L-proline (162 mg/ml), and L-lysine hydrochloride (100 mg/ml). The soluble group has seven amino acids with 50 mg/ml concentration: L-alanine, L-valine, L-isoleucine, L-threonine, L-methionine, L-cysteine, and L-histidine hydrochloride. The sparingly soluble group has four amino acids: L-asparagine (25 mg/ml), L-phenylalanine (25 mg/ml), L-leucine (24 mg/ml), and L-glutamine (18 mg/ml). The slightly soluble group has the remaining four amino acids: L-glutamic acid (8.6 mg/ml), L-aspartic acid (5 mg/ml), L-tyrosine (3.8 mg/ml) and L-tryptophan (3.8 mg/ml). For comparison, we carried out four control experiments without laser irradiation. 30 μl of an aqueous solution of amino acid was placed on the microscope coverslip without any nucleant (Control-NN), with aluminum wire (Control-AL), with peacock feather barbule (Control-PF), and with coconut coir (Control-CO). Laser-induced crystallization experiments were conducted under identical conditions with three nucleants, aluminum (Laser-AL), peacock feather barbule (Laser-PF), and coconut coir (Laser-CO), respectively. The nucleants were placed at or very near the boundary of the air-solution interface. The time taken for the first crystal to appear inside the solution was visualized using at least three different video recordings in each experiment; in many experiments up to ten video recordings were used. The average times taken to observe the first crystals are summarized in Table 1. Within the laser focal volume, initially only a single nucleation event occurs; thereafter, there may be multiple nucleation due to the fast speed at which crystallization occurs in these experiments. However, it has generally not been possible to control the number of nucleation events in the present experiments. We have observed both nucleation clusters as well as single crystals (see the Videos in Supplementary Information).

Single crystal X-ray diffraction data of amino acid crystals were collected using a Rigaku Saturn724+ diffractometer (Mo Kα radiation, λ = 0.71075 Å). The cell parameters of the crystals were verified for selected amino acids (Gly, Ala, Thr, Cys, Asp, Glu, Ser, and His) and compared with the Cambridge Structural Database (CSD). In order to understand the surface morphology of coir, electron microscope (SEM) images were collected.

## Electronic supplementary material

Supplementary Information

Video SV1

Video SV2

Video SV3

Video SV4

Video SV5

## Data Availability

All data generated or analysed during this study are available from the corresponding author on reasonable request.
