# Supplementary material for: Effect of biocompatible nucleants in rapid crystallization of natural amino acids using a CW Nd:YAG laser
Source: Sci Rep. 2018 Oct 30;8:16018. doi: 10.1038/s41598-018-34356-0 (PMC6207789; doi:10.1038/s41598-018-34356-0)
Supplement: Supplementary file 1 — Supplementary Information [file 41598_2018_34356_MOESM1_ESM.docx]

Effect of biocompatible nucleants in rapid crystallization of natural amino acids a using CW Nd:YAG laser

**Shilpa Thippeshappa^1^, Sajan D. George^1,2^, Aseefhali Bankapur^1^, Santhosh Chidangil^1^, Deepak Mathur^1^, Abdul Ajees Abdul Salam^1^***

^1^Department of Atomic and Molecular Physics, Manipal Institute of Technology Campus, Manipal Academy of Higher Education, Manipal 576 104, Karnataka, India. ^2^Centre for Applied Nanosciences, Manipal Institute of Technology Campus, Manipal Academy of Higher Education, Manipal 576 104, Karnataka, India.

***Supplementary Information***

Table ST1. Time taken for the crystallization of polar amino acids given in seconds. The column marked “Fold Increase” quantifies the time acceleration factor for laser-induced rapid crystallization.

| Amino  acid | Laser-AL | Laser-PF | Laser-CO | Average of Laser | Control-NN | Control-AL | Control-PF | Control-CO | Average of Control | Fold Increase |
| --- | --- | --- | --- | --- | --- | --- | --- | --- | --- | --- |
| Gly | 3.3±0.3 | 5±0.8 | 5±1.5 | 4 | 1720±106 | 1320±60 | 1360±53 | 1440±92 | 1460 | 329 |
| Ser | 6.3±0.6 | 7.5±0.5 | 51.5±1 | 22 | 3780±35 | 3760±20 | 3760±5 | 3540±250 | 3710 | 170 |
| Thr | 7.5±2.5 | 4.5±0.5 | 37.7±4.7 | 17 | 3460 ± 174 | 3120±20 | 4000±20 | 3060±60 | 3420 | 206 |
| Cys | 7±0.6 | 4.5±0.5 | 18±11.5 | 10 | 2320±20 | 1920±35 | 2060±122 | 1680±35 | 1995 | 203 |
| Tyr | 8±1.4 | 21±1 | 81±2 | 37 | 2840±140 | 2900±100 | 2760±60 | 2840±72 | 2835 | 77 |
| Asn | 10±1 | 95±2.5 | 75±11 | 60 | 2800±231 | 2800±106 | 3280±144 | 2940±92 | 2955 | 49 |
| Glu | 24±0.5 | 28±1.7 | 84.5±1.7 | 46 | 1480±180 | 1440±20 | 1320±60 | 1380±20 | 1405 | 31 |
| Gln | 14±1.6 | 24.5±1 | 12.7±0.3 | 17 | 4200±183 | 3620±231 | 3160±160 | 3640±80 | 3655 | 214 |
| Asp | 2.5±0.5 | 85±0.8 | 86±2.5 | 58 | 3800±20 | 3640±203 | 3460±140 | 3380±120 | 3570 | 62 |
| His | 84±4.6 | 233±11 | 110±15 | 142 | 4740 ± 104 | 4100± 111 | 4420±100 | 3980±20 | 4310 | 30 |
| Lys | 4.5±0.5 | 25±1 | 22±3 | 17 | 3600±92 | 3660±35 | 2900±280 | 3360±193 | 3380 | 197 |
| Average | 16 | 48 | 53 | 39 | 3158 | 2935 | 2953 | 2840 | 2971 | 76 |

Table ST2. Time taken for the crystallization of non-polar amino acids. The column marked “Fold Increase” quantifies the time acceleration factor for laser-induced rapid crystallization.

| Amino  acid | Laser-AL | Laser-PF | Laser-CO | Average of Laser | Control-NN | Control-AL | Control-PF | Control-CO | Average of Control | Fold Increase |
| --- | --- | --- | --- | --- | --- | --- | --- | --- | --- | --- |
| Ala | 3.3±0.3 | 4.3±0.3 | 15.3±1.5 | 8 | 3760±20 | 3540±6 | 3660±20 | 3600±13 | 3640 | 477 |
| Val | 4.3±0.3 | 8±1 | 26.3±1.7 | 13 | 2220±60 | 1800±60 | 1780±53 | 1660±20 | 1865 | 145 |
| Met | 3.3±0.3 | 6.3±0.3 | 13±0.6 | 8 | 540 ±20 | 520±20 | 520±20 | 500±20 | 520 | 69 |
| Ile | 8.7±0.3 | 32±3 | 12.5± 0.5 | 18 | 620±20 | 560±40 | 540±60 | 540±60 | 565 | 32 |
| Leu | 6 ±0.5 | 88±11 | 8±0.6 | 34 | 520±20 | 460±40 | 500±40 | 440±20 | 480 | 14 |
| Phe | 5±1 | 12±7 | 49.5±2.5 | 22 | 860±20 | 760±80 | 800±40 | 640±40 | 765 | 35 |
| Trp | 20.7±0.6 | 8.5±1.5 | 49±3.2 | 26 | 3760±53 | 3380±20 | 3600±60 | 3380±20 | 3530 | 135 |
| Pro | 11.3±1.8 | 20±8 | 13±1.5 | 15 | 4120±105 | 4240± 80 | 2560±160 | 3960±13 | 3720 | 252 |
| Average | 8 | 22 | 23 | 18 | 2050 | 1908 | 1745 | 1840 | 1886 | 106 |

Table ST3. Cell parameters of the amino acids crystals obtained using single-crystal X-ray crystallography. The α β, and γ are 90° for all amino acids except glycine where γ=120°. The “*” represents data corresponding to the current study, to be compared to corresponding reference data (marked CSD).

| Amino acid | a(Å) | b(Å) | c(Å) | Cell  Volume | System | Space Group | Reported R-factor in CSD (%) |
| --- | --- | --- | --- | --- | --- | --- | --- |
| Glycine^*^ | 7.038(1) | 7.033(1) | 5.474(9) | 234.66 | Trigonal | P3_2_ | 10.8 |
| Glycine:  CSD 1169353 | 7.037 | 7.037 | 5.483 | 235.14 |  |  |  |
| Alanine^*^ | 5.785(9) | 6.018(6) | 12.332(2) | 429.33 | Orthorhombic | P2_1_cn | 5.41 |
| Alanine:  CSD 756487 | 5.8409(9) | 6.043(2) | 12.028(2) | 424.55 |  |  |  |
| Threonine^*^ | 5.141(7) | 7.727(2) | 13.603(9) | 540.37 | Orthorhombic | P2_1_2_1_2_1_ | 6.80 |
| Threonine:  CSD 1208538 | 5.162(2) | 7.753(1) | 13.630(5) | 545.49 |  |  |  |
| Cysteine^*^ | 5.428(4) | 8.102(2) | 12.177(2) | 533.98 | Orthorhombic | P2_1_2_1_2_1_ | 2.09 |
| Cysteine  CSD 683374 | 5.4201(9) | 8.1164(14) | 12.117(2) | 533.07 |  |  |  |
| Aspartic acid^*^ | 5.586(6) | 9.826(10) | 11.787(8) | 646.97 | Orthorhombic | P2_1_2_1_2_1_ | 3.49 |
| Aspartic acid: CSD 1137053 | 5.565(1) | 9.774(2) | 11.698(3) | 636.28 |  |  |  |
| Glutamic acid^*^ | 5.156(4) | 6.932(2) | 17.273(1) | 617.36 | Orthorhombic | P2_1_2_1_2_1_ | 6.00 |
| Glutamic acid:  CSD 1206528 | 5.154(1) | 6.942(1) | 17.274(3) | 618.05 |  |  |  |
| Serine^*^ | 5.622(4) | 8.507(4) | 9.352(2) | 447.27 | Orthorhombic | P2_1_2_1_2_1_ | 5.03 |
| Serine:  CSD 249277 | 5.6140(9) | 8.5886(18) | 9.345(2) | 450.58 |  |  |  |
| Histidine hydrochloride^*^ | 6.842(7) | 8.940(5) | 15.293(2) | 935.43 | Orthorhombic | P2_1_2_1_2_1_ | 7.6 |
| Histidine hydrochloride:  CSD 1176649 | 6.852(1) | 8.9265(8) | 15.297(3) | 935.63 |  |  |  |

Table ST4. Crystal data and structure refinement for single crystals of C_3_H_7_NO_2_S cysteine molecule grown using our laser-induced technique. A suitable crystal was selected and data were collected using a Rigaku Saturn724+ diffractometer. The crystal was kept at room temperature during data collection. Using Olex2^1^, the structure was solved with the olex2.solve^2^ structure solution program using Charge Flipping and refined with the olex2.refine^2^ refinement package using Gauss-Newton minimization. Note that the crystal was grown within a single 30 μl droplet; the tabulated data pertain to an extremely small crystal of size less than 0.1 mm.

| Identification | Cysteine  (present work) | Cysteine  (from CSD  code: 683374) |
| --- | --- | --- |
| Empirical formula | C_3_H_7_NO_2_S | C_3_H_7_NO_2_S |
| Formula weight | 121.16 | 121.16 |
| Temperature/K | 293.15 | 200(2) |
| Crystal system | Orthorhombic | Orthorhombic |
| Space group | P2_1_2_1_2_1_ | P2_1_2_1_2_1_ |
| a/Å | 5.4180(9) | 5.4201(9) |
| b/Å | 8.1002(10) | 8.1164(14) |
| c/Å | 12.1671(17) | 12.117(2) |
| α/° | 90.00 | 90.00 |
| β/° | 90.00 | 90.00 |
| γ/° | 90.00 | 90.00 |
| Volume/Å^3^ | 533.98(13) | 533.07(16) |
| Z | 4 | 4 |
| ρ_calc_g/cm^3^ | 1.5070 | 1.510 |
| μ/mm^‑1^ | 0.491 | Not available (NA) |
| F(000) | 256.6 | NA |
| Crystal size/mm^3^ | 0.1 × 0.1 × 0.1 | NA |
| Radiation | Mo Kα (λ = 0.71073) | NA |
| 2Θ range for data collection/° | 6.04 to 62.48 | NA |
| Index ranges | -6 ≤ h ≤ 7, -11 ≤ k ≤ 11, -16 ≤ l ≤ 17 | NA |
| Reflections collected | 7146 | NA |
| Independent reflections | 1591 [R_int_ = 0.1388, R_sigma_ = 0.0808] | NA |
| Data/restraints/parameters | 1591/0/69 | NA |
| Goodness-of-fit on F^2^ | 1.024 | NA |
| Final R indexes [I>=2σ (I)] | R_1_ = 0.0665, wR_2_ = 0.1666 | R_1_ = 0.0209, wR_2_ = 0.0579 |
| Final R indexes [all data] | R_1_ = 0.0958, wR_2_ = 0.1973 | R_1_ = 0.0219, wR_2_ = 0.0584 |
| Largest diff. peak/hole / e Å^-3^ | 0.42/-0.66 | NA |
| Flack parameter | -0.18(16) | -0.10(8) |

**References:**

1. Dolomanov, O.V., Bourhis, L.J., Gildea, R.J, Howard, J.A.K. & Puschmann, H. (2009), J. Appl. Cryst. 42, 339-341.
2. Bourhis, L.J., Dolomanov, O.V., Gildea, R.J., Howard, J.A.K., Puschmann, H. (2015). Acta Cryst. A71, 59-75.

**Video clips**

Video SV1. Real-time movie clip showing the time evolution of serine crystals under laser irradiation.

Video SV2. Real time rapid growth of alanine crystals when 1064 nm laser was incident on a peacock feather barbule.

Video SV3. Real time rapid growth of leucine crystals when 1064 nm laser was irradiated on coir.

Video SV4. Real time rapid growth of aspartic acid crystals upon the irradiation by 1064 nm laser on Al wire.

Video SV5. Real time movie clip showing bubble formation and collapse during laser exposure of alanine in the presence of aluminum nucleant. The formation is mostly beneath the aluminum wire and initiates crystallization. Upon reaching large enough size, the bubbles collapse and the associated violence leads to break-up of the crystal.
